# Intestinal Obstruction in a Colt

**Published:** 1896-07

**Authors:** R. P. Lyman

**Affiliations:** Hartford, Conn.


### REPORTS OF CASES.

### INTESTINAL OBSTRUCTION IN A COLT.

By R. P. LYMAN, M.D.V.,
HARTFORD, CONN.

My attention was recently occupied by a case which may possibly be of interest to readers of the Journal, and one which I am led to consider of rather rare occurrence. I was summoned hurriedly to attend a colt, not yet three weeks old, that was exhibiting severe signs of abdominal pain.

On my arrival I found the little fellow prostrated, apparently too weak to rise, breathing heavily, and covered with a cold sweat. On inquiry I learned that he had not done well since he was foaled, although he had never been backward about feeding; he was dumpish, thin, and had a dull, staring coat. Just previous to my arrival he had been very violent in his demonstrations of abdominal disorders, biting his flanks, rolling, crying, sweating, and, in short, expressing all colicky symptoms. By examination and history I diagnosed a stoppage of the bowels, accompanied with a collection of abdominal fluid. Prognosis was unfavorable, in fact he was then in a dying condition. Treatment was administered to no avail, the colt dying in half an hour.

Upon post-mortem, when I opened the abdomen a considerable quantity of greenish-yellow fluid, holding in suspension yellowish flaky particles, was liberated. The bowels were injected throughout; tracing toward the anterior I encountered an enlargement which proved to be a portion of intestine that had telescoped within another; anterior to this was a large collection of fecal material encased in the bluish-black bowel and presenting anteriorly considerable gangrene of the intestine leading from the stomach. Upon the telescoped portion I incised a watery cyst about the size of a hen's egg and entirely obstructing the alimentary passage.

The case showed evidences of extended duration, and, coupled with its history, it is fair to suppose the pathological condition was of congenital origin suddenly made acute by the accumulated alimentary matters.

